# Prevalence and outcome of COVID-19 among Iranian celiac patients 

**Published:** 2022

**Authors:** Fahimeh Sadat Gholam-Mostafaei, Nastaran Asri, Naser Parvani, Elham Aghamohammadi khamene, Farnoosh Barzegar, Mohammad Rostami-Nejad, Mostafa Rezaei-Tavirani, Bijan Shahbazkhani, Somayeh Jahani-Sherafat, Kamran Rostami, Mohammad Reza Zali

**Affiliations:** 1 *Gastroenterology and Liver Diseases Research Center, Research Institute for Gastroenterology and Liver Diseases, Shahid Beheshti University of Medical Sciences, Tehran, Iran*; 2 *Basic and Molecular Epidemiology of Gastrointestinal Disorders Research Center, Research Institute for Gastroenterology and Liver Diseases, Shahid Beheshti University of Medical Sciences, Tehran, Iran*; 3 *Proteomics Research Center, Faculty of Paramedical Sciences, Shahid Beheshti University of Medical Sciences, Tehran, Iran*; 4 *Department of Gastroenterology and Liver Diseases, Imam Khomeini Hospital Complex, Tehran University of Medical Sciences, Iran*; 5 *Laser Application in Medical Sciences Research Center, Shahid Beheshti University of Medical Sciences, Tehran, Iran*; 6 *Department of Gastroenterology Mid-Central District Health Board, Palmerston North, New Zealand*

**Keywords:** Covid-19, Celiac disease, SARS-CoV-2, Infections

## Abstract

**Aim:**

This study aimed to evaluate the prevalence and outcome of COVID-19 among Iranian celiac disease patients.

**Background:**

Patients with celiac disease (CD) might be at greater risk for opportunistic viral infections. Coronavirus disease-2019 (COVID-19) is a new coronavirus (SARS-CoV-2) cause of respiratory disorder which spread around the world at the end of 2019. The question is does COVID-19 infection increase the risk of severe outcome and/or a higher mortality in treated celiac disease?.

**Methods:**

Data regarding demographic details, clinical history, and COVID-19 infection symptoms among treated celiac disease patients was collected from July 2020 to January 2021 and analyzed using SPSS version 25.

**Results:**

A total of 455 celiac disease patients were included in this study. The prevalence of Covid-19 infection among celiac disease patients was 2.4%. Infection among women (72.7%) was higher than the men, and only one overweight man who smoked was hospitalized. Among COVID-19 infected celiac disease patients, the most common symptoms were myalgia 90.9% (10/11), fever, body trembling, headache, shortness of breath, loss of smell and taste, and anorexia (72.7%). Treatments for COVID-19, included antibiotics (90.9%), pain analgesics (54.5%), antihistamines (27.3%), antivirals (9.1%) and hydroxychloroquine (9.1%).

**Conclusion:**

This study shows that treated celiac disease is not a risk factor for severity or higher mortality in patients infected with COVID-19. Women, however, might need extra-protection to prevent COVID-19 infection.

## Introduction

 Celiac disease (CD) is a lifelong autoimmune disorder of the small intestine caused by the ingestion of gluten-containing foods in genetically susceptible subjects (HLA DQ2 and/or DQ8 haplotypes carriers (1, 2). Those with treated CD have a similar life expectancy and resistance to external pathogens as the general population. 

Coronavirus disease 2019 (COVID-19), caused by severe acute respiratory syndrome coronavirus 2 (SARS-CoV-2), first broke out in China at the end of 2019 and then spread to the whole world and became a pandemic in March 2020. The clinical symptoms of COVID-19 are similar to those of the flu and can be accompanied only by gastrointestinal (GI) presentations (3). Old age, pulmonary and heart disease, cancer, diabetes, hypertension, viral infection such as hepatitis B, chronic renal disease, and immune-deficiency are risk factors for severe complications of COVID-19 (4). Studies have reported an association between increased risk of severe COVID-19 infection, high mortality rate, and immunodeficiency diseases in adults (5, 6). Conversely, Emmi et al. reported that the risk of COVID-19 infection in people with systemic autoimmune diseases is not more than that of the general population (7). It has been reported that CD patients may be at greater risk of infection with viral illnesses and developing severe respiratory complications compared to the general population (8, 9). On the other hand, in studies conducted by Gokden and Zingone et al., it was found that there is no increased risk of COVID-19 infection complications in CD patients on a gluten-free diet (GFD) compared to non-celiac individuals (10, 11). The limited and partially contradictory data related to COVID-19 complications and mortality in CD has created concerns among CD patients and health professionals. In the current study, we conducted a questionnaire-based analysis, aiming to evaluate the prevalence and outcomes of COVID-19 in Iranian celiac disease patients. 

## Methods

This cross-sectional study was designed by the Celiac Department of the Research Institute of Gastrointestinal and Liver Disease at Taleghani Hospital, Tehran, Iran, to investigate the risk of Covid-19, its complications and its mortality rate among adult CD patients (≥18 years). From July 2020 to January 2021, we contacted all CD patients in our CD registry and had been treated with a gluten-free diet for at least six months. The study was approved by the Research Ethics Committee of the Research Institute for Gastroenterology and Liver Diseases (RIGLD, Shahid Beheshti University of Medical Sciences, Tehran, Iran, IR.SBMU.RETECH.REC.1399.088). Patients were asked if they had been infected with COVID-19, and our counselors gave them healthcare guidelines and advised them to stay home as much as possible and avoid unnecessary visits to medical centers. 

Included in this study were 455 CD patients, all of whom were diagnosed based on serological and histopathological criteria. COVID-19 infection was detected by positive RT-PCR or chest computed tomography (CT) scan results for SARS-CoV-2. Data regarding demographic details, clinical history, COVID-19 symptoms, and diagnostic tests was recorded. The survey also investigated the presence of other underlying diseases, considering that they can lead to a worse outcome in COVID-19 infection. 

Statistical analyses were carried out using SPSS version 25 (IBM, Armonk, NY), and *p*-values less than 0.05 were deemed to be significant. 

## Results

Overall, out of the 455 CD patients who enrolled in this eight-month follow-up study, 11 (2.4%) participants reported having a COVID-19 diagnosis. The median age of the participants was 35 years, and 314 (69%) participants were women. The median BMI of patients was 22.20; 15% of participants were underweight, 48.3% had normal weight, and 31.2% and 5.4% were overweight and obese, respectively (according to World Health Organization criteria). Most COVID-19 infected patients were of normal weight, except two participants who were overweight and one who was obese. None of the patients were diagnosed with refractory celiac disease type 1 or type 2. Body mass index (BMI) was over 25 in four (36.4%) patients with COVID-19, and most (3/4) of them were men.

Women (72.7% (8/11)) were more likely to be infected with COVID-19 than men (27.3% (3/11)), but no significant relationship was found between gender and COVID-19 infection (8 out of 314 women with celiac disease (2.54%) and 3 out of 141 men (2.12%) became infected with COVID-19; *p*-value=0.78). Myalgia (90.9% (10/11)) was the most frequent symptom in CD cases that presented in 100% of men and 87.5% of women with COVID-19. The six most common symptoms among patients with COVID-19 were fever, body trembling, headache, shortness of breath, loss of smell and taste, and anorexia. Fever, loss of smell and taste, body trembling, shortness of breath and anorexia were more common in men, and headache, sore throat, and vomiting were more common in women (Table 1). Although the CT scan results of 2 patients showed lung abnormalities, only one of them was hospitalized (an overweight (BMI=29.5) 56-year-old man who smoked). About 27.3% (3/11) of COVID-19 infected patients had another chronic disease such as diabetes type 2 (18.2%), cardiovascular disease (9.1%), thyroid dysfunction (9.1%), a liver disorder (9.1%), or a neurological disorder (9.1%). 

**Table 1:** Signs and symptoms among CD patients with confirmed COVID-19 infection

Total frequency in CD patients %(n) N=11	Frequency in women%(n) N=8	Frequency in men %(n) N=3	Covid-19 symptom
72.7(8/11)	62.5(5/8)	100 (3/3)	Fever
72.7(8/11)	62.5(5/8)	100 (3/3)	body trembling
0	0	0	Consciousness
45.4(5/11)	37.5(3/8)	66.7 (2/3)	Cough
72.7(8/11)	75(6/8)	66.7 (2/3)	Headache
72.7(8/11)	62.5(5/8)	100 (3/3)	Shortness of breath
90.9 (10/11)	87.5(7/8)	100 (3/3)	Myalgia
45.4(5/11)	50(4/8)	33.3 (1/3)	Sore throat
36.4(4/11)	25 (2/8)	66.7 (2/3)	Chest pain
72.7(8/11)	62.5(5/8)	100 (3/3)	Loss of Smell and taste
27.3(3/11)	25 (2/8)	33.3 (1/3)	Diarrhea
36.4(4/11)	37.5(3/8)	33.3 (1/3)	Vomiting
45.4(5/11)	37.5(3/8)	66.7 (2/3)	Stomachache
72.7(8/11)	62.5(5/8)	100 (3/3)	Anorexia
9.1(1/11)	0	33.3 (1/3)	Digest bleed

**Figure1:**
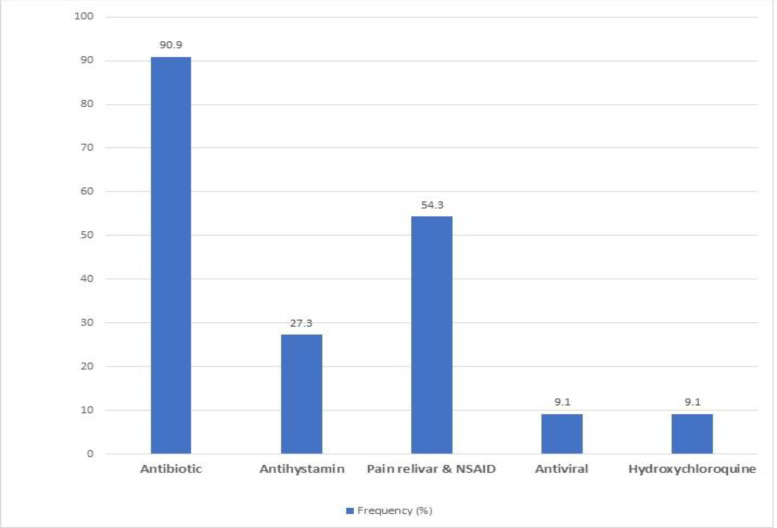
Medication used for COVID-19 infection. Antibiotics: ceftriaxone, azithromycin, cefixime Antihistamine: dimenhydrinate, cetirizine, and diphenhydramine; Pain reliever & NSAID: acetaminophen and naproxen, Antiviral: remdesivir

The oral macrolide antibiotic azithromycin was widely prescribed (72.7%) to treat the bacterial suprainfection in COVID-19. Acetaminophen (36.4%) and naproxen (18.2%) were also prescribed to relieve pain and fever. With lesser frequency, ceftriaxone and cefixime, two cephalosporin antibiotics, were used to treat respiratory infections, and other drugs such as hydroxychloroquine, cetirizine, diphenhydramine, and dimenhydrinate were used to improve new coronavirus symptoms (Figure 1). Pharmaceutical care for the patient hospitalized for seven days included ceftriaxone and remdesivir. All cases reported that they had good hygiene and washed their hands for at least 20 seconds; furthermore, they used personal protective equipment such as masks and gloves during their COVID-19 infection. However, quarantine was not properly completed in 63.6% of patients. The incomplete variables were therefore deleted from the study design.

## Discussion

The COVID-19 pandemic has placed a heavy burden of anxiety and stress on patients with chronic diseases. Moreover*, *little is known about the prevalence of COVID-19 infection in some gastrointestinal disorders such as CD. This study aimed to evaluate the susceptibility of GFD-treated CD patients to novel coronavirus infection. The critical finding of the current study was that the incidence of COVID-19 infection among CD patients was 2.4%, with a predominance in female gender. This may point to the female gender having a higher affinity for contracting COVID-19 infection among CD. Other studies have reported that the prevalence of COVID-19 in men was higher than women (12, 13); however, hospitalization and severe outcome of Covid-19 were not increased among the studied CD patients. The results of a recently-published study on the risk of severe COVID-19 among Swedish CD patients were similar to those of the current study (14). In the present study, only a 56-year-old, overweight, male smoker was hospitalized for seven days because of COVID-19. Evidence shows that both smoking and higher BMI are two risk factors for different respiratory infections and diseases, including COVID-19. In fact, the expression of angiotensin-converting enzyme 2 (ACE2) is increased in lung epithelial cells and adipose tissue due to obesity and smoking, which leads to increased risk of severe COVID-19 infection (15, 16). A recent population-based cohort study found that 0.14% (58/40,963) of COVID-19 infected CD individuals required hospitalization (14). A single-center epidemiological study in Baqiyatallah Hospital in Tehran, Iran, conducted from 19 February 2020 to 15 April 2020, reported that about 23% of total COVID-19 patients (without CD) who visited the emergency department were hospitalized (12). Their findings also suggested that high BMI appeared to play a key role in the COVID-19 prognosis of celiac patients. Results on BMI and increased risk of COVID-19 were similar to those of previous studies showing a significant relationship between higher BMI and more severe COVID-19 (15, 17). Individuals infected with COVID-19 and having a higher BMI are at higher risk for hospitalization, intensive clinical care requirements, and death. Although COVID-19 infection is mainly known to have respiratory symptoms varying from mild to severe, myalgia was the most common COVID-19 symptom observed in our CD patients, and this finding was consistent with what was observed in the general population (18). Other studies from Wuhan, however, have reported that fever and cough were more common in their studied patients (19, 20)*. *Diabetes mellitus is a metabolic disorder, and diabetic patients are at increased risk of respiratory infection (21). Evidence shows that type 2 diabetes predisposes CD patients to a more severe outcome (22). Initial research found that increased risk of COVID-19 infection and its severity in diabetic patients might contribute to hyperglycemia in them (23). Accordingly, our results showed that 18.2% (2/11) of the studied CD patients who became infected with COVID-19 were diabetic. However, disease severity was not such that any of them required hospitalization. Although the FDA has not approved antibacterial treatment for COVID-19, like azithromycin, this antibiotic was widely used to fight bacterial suprainfection in the treatment protocol of these patients (72.7%). Several studies suggest that azithromycin cannot be considered as an effective treatment for COVID-19-related supra-infection (24, 25). In conclusion, this study reassuringly shows that treated CD patients were not at increased risk of severe COVID-19 infection or hospitalization compared with the general population. The female gender had a higher prevalence of COVID-19. This may suggest that female CD patients might need extra protection against contracting COVID-19. The current study evaluated only treated CD patients on a gluten-free diet. Further studies are needed to investigate the COVID-19 prognosis and complications in active CD patients.

## Conflict of interests

The authors declare that they have no conflict of interest.
